# The cross-cultural adaptation and psychometric properties of the Graded Chronic Pain Scale-Revised—Simplified Chinese version

**DOI:** 10.1371/journal.pone.0292747

**Published:** 2023-10-10

**Authors:** Bing Liang, Yuejin Wu, Jiaxin Zhang, Shumin Hao, Feng Li

**Affiliations:** School of Nursing, Jilin University, Changchun, China; Mugla Sitki Kocman Universitesi, TURKEY

## Abstract

Chronic pain is a prevalent issue worldwide and is a significant contributor to human suffering and disability. The Graded Chronic Pain Scale-Revised has exhibited favorable reliability and validity. However, its applicability yet to be explored in China. We aimed to create a simplified Chinese version of the Graded Chronic Pain Scale-Revised for chronic pain patients by conducting cross-cultural adaptation and psychometric evaluation. This study employs a two- phase design. In phase 1, the Graded Chronic Pain Scale-Revised was cross-culturally translated and adapted in accordance with international guidelines. In phase 2, the simplified Chinese version of the Graded Chronic Pain Scale-Revised was administered to 417 participants along with Numerical Rating Scale to assess its psychometric properties. The final analysis consisted of data from 376 participants. The scale had a Cronbach’s α coefficient of 0.944. Moreover, the scale exhibited excellent content validity and was divided into two dimensions: identifying high impact chronic pain; and the Pain, Enjoyment, and General Activities subscale. Exploratory and confirmatory factor analyses revealed that these dimensions had a good model fit. Additionally, the simplified Chinese version of the Graded Chronic Pain Scale-Revised demonstrated good convergent and discriminant validity. The receiver operating characteristic curve demonstrated that grades 2 and 3 had a good predictive effect on limiting participants’ work ability, and the area under the receiver operating characteristic curve was equal to 0.91. The present study demonstrates the successful adaptation of the Graded Chronic Pain Scale-Revised into Simplified Chinese, with the revised version exhibiting favorable psychometric properties. This scale addresses the shortcomings of domestic chronic pain grading assessment tools, providing a valuable instrument for evaluating the severity of chronic pain in Chinese clinical practice and serving as a reference and basis for other research related to chronic pain.

## 1 Introduction

According to the International Association for the Study of Pain (IASP), chronic pain is defined as pain that lasts for three months or longer, exceeding the normal healing time of tissues [1, 2]. Regardless of whether it is caused by illnesses, chronic pain does not immediately endanger human life, but many people have to live with it [3, 4]. It is a major source of human suffering and disability [5]. In the United States, pain has been identified as a major public health problem, with 50 million adults experiencing chronic pain and 20 million adults suffering from severe pain [5]. A systematic review found that the prevalence of chronic pain among Asian adults ranged from 7.1% (Malaysian adults) to 90.8% (elderly Chinese adults) [6]. Long-term pain not only causes patients great physical pain and seriously affects their quality of life but also causes psychological or mental distress. Anxiety and depression are reported to be very common among chronic pain patients [7]. The dual burden of physical and psychological distress may lead to poor health outcomes for patients as well as increases in personal and societal health care costs and burdens [8]. Therefore, early identification of chronic pain and strengthening symptom management are necessary. In the treatment of chronic pain patients, pain assessment has been described as a fundamental aspect of pain management, and it plays a crucial role in nursing [9]. Scientific pain assessment tools are key to effective pain treatment, and clarifying the pain grade of the tested subjects is one of the prerequisites for targeted and efficient treatment.

Many studies have investigated the effectiveness of chronic pain assessment tools. Among these tools, the Visual Analog Scale (VAS) remains the gold standard for chronic pain assessment [10]. It can be used to measure not only the intensity of pain, but also the degree of pain relief [11]. Its reliability ranges from 0.71 to 0.94, and its validity ranges from 0.62 to 0.91 [12], thus demonstrating its effectiveness and stability. This pain assessment method was introduced in China in the past century. However, despite being a simple and effective pain assessment tool, it may be influenced by different factors, such as age, gender, and personality traits [13]. Additionally, its item settings are relatively abstract, and the response rate is lower in older adults [14]. Therefore, the VAS is not suitable for patients with low education levels or cognitive impairment. Other commonly used chronic pain assessment tools include the Numerical Rating Scale (NRS), which has a reliability of up to 0.95 and a validity greater than 0.86. The advantages of the NRS include its simplicity and intuitiveness. It is currently used in populations of all ages [12].

The Graded Chronic Pain Scale (GCPS) was developed by Michael Von Korff in 1991 [15]. Based on data from 2389 pain patients, this scale was developed using chronic pain-relevant indicators and epidemiological research methods to evaluate the severity of patients’ chronic pain. The scale assesses pain severity and pain-related disability on a scale ranging from grades 0 to 4 [15]. The GCPS has good reliability and validity in evaluating pain grades [15], and has shown advantages over other pain scales because it measures health-related outcomes more comprehensively [16]. The GCPS was originally developed in English and tested in several English-speaking populations, showing good reliability and validity [17–21]. In addition, it has been successfully adapted to Arabic [16], Spanish [22], Turkish [23], and other languages. However, chronic pain patients usually have multiple pain sites [24], and the essence of the GCPS is to evaluate pain status based on anatomical definitions [15]; therefore, it cannot be used to comprehensively evaluate patients’ pain from a biopsychosocial perspective. Moreover, because the GCPS uses two time frames (3 months and 6 months) to evaluate subjects’ pain grades on a scale of 0–10, the scoring rules are relatively complex [25]. Therefore, researchers have further developed and revised the GCPS to enhance its practicality.

In 2019, Michael Von Korff revised the GCPS to create the Graded Chronic Pain Scale-Revised (GCPS-R), which can be used to identify chronic pain patients and differentiate the severity of chronic pain (mild, bothersome, and high-impact chronic pain) [25]. The scale was initially validated in a sample of 2021 adults and strongly predicted five indicators of activity limitations, with a receiver operator curve of 0.76 to 0.89, providing a simpler and more effective way to evaluate chronic pain [25]. Since its development, it has been translated into Spanish and Turkish [26, 27] with good reliability and validity [23], while a simplified Chinese version has not yet been developed and validated.

In this study, we aimed to analyze the psychometric properties of the Graded Chronic Pain Scale-Revised—Simplified Chinese version (C-GCPS-R) and explore its applicability in the Chinese population. This work can provide a reliable tool for Chinese clinical staff to assess chronic pain promptly, accurately, and effectively. This tool can be used to grade the pain status of Chinese chronic pain patients and develop personalized interventions based on different pain grades to reduce patients’ pain symptoms, ultimately improving their quality of life and preventing adverse outcomes.

## 2 Methods

### 2.1 Study design

This study utilized a two-phase design. In the first phase, the GCPS-R was translated and cross-culturally adapted to develop the simplified Chinese version [25], using the cross-cultural adaptation procedures recommended by Brislin [28] and Jones [29], and to conduct a pretest. Feedback from participants was used to modify the scale’s wording, phrasing, structure, and other relevant aspects. In the second phase, a cross-sectional study was conducted to evaluate the psychometric properties of the C- GCPS-R. Reliability was analyzed using internal consistency, while validity was analyzed using multiple indicators. In addition, the study employed the ROC to calculate the optimal cutoff score, to examine the impact of pain severity on work, and to assess its predictive accuracy.

### 2.2 Phase I: Translation and cross-cultural adaptation of the GCPS-R

#### 2.2.1 Permissions

Authorization was obtained from Professor Michael Von Korff, the author of the GCPS-R, via email.

#### 2.2.2 Translation and back translation

The translation and back-translation of the scale was conducted using Brislin’s intercultural translation model [30], which involved inviting two bilingual medical English teachers (T1 and T2) to independently translate the scale. The research team then combined the two translation versions, discussed any inconsistencies with T1 and T2, and developed the initial C-GCPS-R1. In the back-translation stage, another bilingual medical English teacher (T3) and an English-speaking medical English expert (T4) were invited to independently translate the C-GCPS-R1 into English. The research team reviewed, organized, and revised the GCPS-R and the two back-translated versions, eliminating any inconsistencies in meaning and terminology, thus yielding the C-GCPS-R2. To ensure translation quality, none of the three medical English teachers or the medical English expert were involved in this study, nor were they informed in advance that they would be asked to undertake any translation work. Finally, cultural adaptation was conducted to ensure the specificity and effectiveness of the C-GPS-R2. A cultural adaptation team consisting of seven experts with clinical experience was invited to participate in this study. The selection criteria for experts were as follows: 1) research areas included basic or rehabilitative nursing, rehabilitation medicine, pain-related specialties, or clinical practice; 2) familiar with related research fields at home and abroad; 3) possessing intermediate or higher professional technical titles, as well as a master’s degree or above; 4) engaged in this field for more than 8 years; and 5) having a certain degree of enthusiasm for this study and complying with the principles of voluntary and informed consent. Without violating the original scale, the cultural adaptation team of experts modified and integrated the items, meanings, and expressions of the scale through expert inquiry forms issued by the research team, making the language conform to Chinese expression habits. The C-GCPS-R3 was further revised by the research team and can be completely interchangeable with the original scale in terms of concept and meaning.

#### 2.2.3 Content validity

The content validity index (CVI) comprises the item-level CVI (I-CVI) and the scale-level CVI (S-CVI). The primary objective is to assess the relevance, representativeness, and appropriateness of the content and measurement range of the items. The content validity experts, along with the cross-cultural adaptation experts, rated the association of each item in the C-GCPS-R3 with chronic pain patients using a four-point rating questionnaire, ranging from "not related" to "strongly related", represented by the numbers 1–4. The I-CVI was determined by calculating the percentage of experts who scored the items as 3 or 4, while the S-CVI was determined by calculating the mean of the I-CVI. Additionally, the item-total correlation was used to test the correlation between the items. An item-total correlation coefficient of less than 0.40 indicates a weak correlation between the item and the overall scale, and such value indicate that the item should be considered for deletion.

#### 2.2.4 Pretest

After determining the C-GCPS-R3, we conducted a pretest using this scale. By using a convenience sampling method, we selected a sufficient number of chronic pain patients who met the inclusion and exclusion criteria from Changchun City, Jilin Province, China, and distributed the C-GCPS-R3 to participants while soliciting feedback on the readability and accuracy of the items. Based on the participants’ feedback, we modified the items’ expression, wording, and structure to form the C-GCPS-R4.

Following the inclusion and exclusion criteria (consistent with the cross-sectional inclusion and exclusion criteria), we randomly selected 30 participants for a pretest (not participating in the cross-sectional study) to determine whether they could understand the meaning of each item and complete the scale correctly. During the testing process, the participants’ understanding of the scale corresponded with the scale’s expression content, and there were no unclear items or semantic ambiguities. The time required to complete the scale was approximately 2–3 minutes, and no changes to the scale were necessary. Therefore, following cross-cultural adaptation, the committee approved the final version of the C-GCPS-R (see S1 File).

### 2.3 Phase 2: Psychometric validation

#### 2.3.1 Ethical considerations

This study was conducted as cross-sectional research, which took place in Changchun, Jilin Province, China from 11/4/2023 to 20/6/2023. All experiments were performed in accordance with the Declaration of Helsinki. Ethical approval was obtained by the Human Research Ethics Committee of the School of Nursing, Jilin University (HREC2023041001). All participants signed written informed consent forms, and the participant information involved was kept confidential by the research team. Additionally, it has been duly registered with the Chinese Clinical Registration Center under the registration number ChiCTR2300070399.

#### 2.3.2 Participants

Upon approval by the Human Research Ethics Committee of the School of Nursing, Jilin University (HREC2023041001), the study participants were recruited from two tertiary comprehensive hospitals. A total of two cohorts were recruited.

The inclusion criteria for patients were as follows: 1) participants were able to express themselves without hearing impairment or communication impairment; and 2) participants were fully informed and willing to participate in this study. The exclusion criteria were as follows: 1) participants with advanced disease; and 2) participants with known severe mental illness and those receiving emergency or interventional surgery. All participants agreed to participate and signed written informed consent forms.

#### 2.3.3 Instruments

The research team, in conjunction with previous studies on the GCPS-R [25] and the epidemiology of chronic pain, measured general characteristics using a structured questionnaire that included 14 items such as age, gender, ethnicity, education level, occupation, smoking status, and opioid drug use. In addition to this, participants were assessed using two instruments, the currently developed the C-GCPS-R and the NRS.

#### 2.3.4 Graded Chronic Pain Scale-Revised—Simplified Chinese version

The C-GCPS-R comprises six items. To identify individuals with chronic pain, item 1 assesses the frequency of pain experienced in the past three months (never, some days, most days, everyday). If item 1 is marked "never" or " some days ", the pain grade is classified as 0 (chronic pain absent). To identify participants with grade 3 pain, participants are asked about the frequency of pain that has limited their activities or work in the past three months, which constitutes item 2 (never, some days, most days, everyday). If item 1 is marked as ’most days’ or ’every day’ and item 2 is marked the same, the pain is classified as grade 3 with high-impact chronic pain (HICP). After identifying chronic pain, items 3 to 5 are evaluated, which constitute the Pain, Enjoyment, and General Activity (PEG) scale. These items assess the severity of pain experienced in the past seven days, with each item scored from 0 to 10. Item 3 evaluates the severity of the pain, Item 4 evaluates the degree to which pain affects enjoyment of life, and Item 5 assesses the extent to which the pain affects daily activities. The total PEG score ranges from 0 to 30, with a score below 12 indicating grade 1 (mild chronic pain) and a score of 12 or higher indicating grade 2 (bothersome chronic pain). Item 6 assesses whether the participant is unable to work or has stopped working due to pain, with "yes" or "no" answers.

#### 2.3.5 Numeric rating scale

The scale utilized in this study requires participants to assess their pain severity [31] using a numerical value ranging from 0 to 10, with 0 indicating no pain and 10 indicating extreme pain [32]. This method has been widely applied across various age groups and boasts a reliability coefficient of 0.95 and a validity coefficient greater than 0.86 [12]. One study showed a high correlation coefficient between the VAS and the NRS [33], with a correlation coefficient ranging from 0.77 to 0.91. The scale has been extensively implemented in clinical practice in China and has been demonstrated to be both reliable and effective. Given the relatively advanced age of the participants recruited for this study, the NRS was deemed a more intuitive tool for assessing pain intensity than the VAS [34]. Participants were able to easily understand and interpret the results of the NRS.

### 2.4 Data collection

The head of the research team provided uniform training to the researchers, ensuring their proficiency in using and scoring with the C-GCPS-R. The first author supervised and directed the participants throughout the scale completion process, with relevant personnel available to clarify any uncertainties. The scales were distributed and collected on-site, with issues such as incomplete or missing submissions addressed immediately. Prior to data entry, the collected scales were carefully reviewed and any unsatisfactory submissions were removed, including those that were incomplete, scored uniformly, had excessive identical selections, or contained logically inconsistent responses.

### 2.5 Statistical analysis

Data entry was conducted independently by two individuals using Epidata 3.1 software to ensure accuracy. Data analysis was performed using SPSS 26.0 and AMOS 26.0 statistical software. Descriptive statistics were used to describe participant characteristics. Sample 1 was used for exploratory factor analysis (EFA), while sample 2 was used for confirmatory factor analysis (CFA).

Internal consistency reliability was used to evaluate the reliability of the C-GPS-R. Cronbach’s α is the most commonly used internal consistency reliability indicator, and a Cronbach’s α value of >0.8 indicates good scale reliability. Additionally, Cronbach’s α was calculated for each dimension, with values greater than 0.8 indicating good reliability.

EFA and CFA were used to evaluate multiple validity indicators of the scale. The Kaiser‒Meyer‒Olkin (KMO) test [35] was first used to determine whether the sample size was sufficient for factor analysis. A KMO value closer to 1.0 indicates better factor analysis results, while values below 0.6 suggest that factor analysis is not appropriate. The Bartlett sphericity test [36] was also conducted to determine whether there were strong correlations between variables in the scale. If P < 0.05, the hypothesis of independence between variables in the scale is not supported, indicating strong correlations. Principal component analysis was used for EFA, with maximum orthogonal variance rotation used to extract common factors. The common factor needed to have a cumulative variance contribution rate > 40%, and each item in the scale needed to have a factor load on the common factor of >0.4 to reflect basic information on a dimension of the scale. CFA was used to evaluate the fit of the model, with evaluation indicators including the chi-square freedom ratio (X^2^/df), root mean square residual (RMR), goodness-of-fit index (GFI), comparative fit index (CFI) and root mean square error of approximation (RMSEA). Additionally, composite reliability (C.R.) and average variance extracted (AVE) were used to evaluate the convergent validity of the C-GCPS-R. AVE values of ≥0.5 and C.R. values of ≥0.7 are considered acceptable [37]. Discriminant validity was evaluated by conducting correlation analysis and extracting the square root of AVE, with the square root of AVE for each factor should be greater than the correlation coefficient between that factor and other factors, with a recommended value of AVE ≥0.5 and correlation values ≤0.85 for each factor.

Criterion validity is a measure of the relationship between the adapted scale and the selected criterion scale, and it is generally expressed by a correlation coefficient to indicate the degree of similarity between the two scales [38]. A correlation coefficient greater than 0.7 is considered acceptable. To examine the relationship between the C-GCPS-R and the NRS, the criterion validity was evaluated by analyzing the scores of 60 randomly selected participants in Sample 2, which corresponds to 10–20% of the total sample size.

## 3 Results

### 3.1 Participant characteristics

The study involved 417 eligible participants, with sample 1 consisting of 100 individuals and sample 2 consisting of 317 individuals. Of these 417 participants, 376 (with sample 1 consisting of 78 individuals and sample 2 consisting of 298 individuals) provided informed consent and completed the scales in their entirety. Participants indicated that the scales were clear and that the items were well answered. Table 1 summarizes the demographic and clinical characteristics of the participants (Table 1).

**Table 1:** Sample characteristics (n = 376).

Variable		Total(n = 376)	Sample 1(n = 78)	Sample 2(n = 298)
**Age (years), N(%)**				
	<18	4	0(0.0)	4(1.3)
	18–60	268	40(51.3)	228(76.6)
	>60	104	38(48.7)	66(22.1)
**Sex, N(%)**				
	Male	168	36(46.2)	132(44.2)
	Female	208	42(53.8)	166(55.8)
**Ethnicity, N(%)**				
	Han	358	70(89.7)	288(96.6)
	Other	18	8(10.3)	10(3.4)
**Education level,N(%)**				
	Elementary school or lower	80	30(38.5)	50(16.8)
	Secondary or technical schools	189	38(48.7)	151(50.7)
	Bachelor or above	107	10(12.8)	97(32.5)
**Marital status, N(%)**				
	Unmarried	76	4(5.1)	72(24.2)
	Married	287	73(93.6)	214(71.8)
	Divorced	7	0(0.0)	7(2.3)
	Bereaved	6	1(1.3)	5(1.7)
**Occupation, N(%)**				
	Unemployed	67	15(19.2)	52(17.4)
	Retirement	91	25(32.1)	66(22.1)
	Manager	12	0(0.0)	12(4)
	Technician	21	0(0.0)	21(7.0)
	Employee	24	2(2.6)	22(7.3)
	Transporter	3	1(1.3)	2(0.7)
	Service personnel	32	24(30.8)	8(2.7)
	Farmer	20	0(0.0)	20(6.7)
	Worker	12	0(0.0)	12(4.0)
	Teacher	6	1(1.3)	5(1.7)
	Student	39	3(3.8)	36(12.1)
	Healthcare Worker	33	2(2.6)	31(10.4)
	Other	16	5(6.4)	11(3.9)
**Smoking, N(%)**				
	Yes	86	18(23.1)	68(22.8)
	No	290	60(76.9)	230(77.2)
**Long-term use of opioids, antidepressants, etc., N(%)**				
	Yes	26	8(10.3)	18(6.4)
	No	350	70(89.7)	280(93.6)
**Drinking, N(%)**				
	Yes	81	13(16.7)	68(22.8)
	No	295	65(83.3)	230(77.2)
**Receiving medical care (within one year), N(%)**				
	Yes	158	49(62.8)	109(36.6)
	No	218	29(37.2)	189(63.4)
**Chronic diseases, N(%)**				
	Yes	99	3544.9)	64(21.5)
	No	277	43(55.1)	234(78.5)
**Ways of medical payments, N(%)**				
	Publicly funded	8	1(1.3)	7(2.3)
	Medical insurance	239	36(46.2)	203(68.1)
	New Cooperative Medical System	101	41(52.6)	60(20.1)
	One’s Own Expense	28	0(0.0)	28(9.5)
P**ain location (within seven days), N(%)**				
	None	111	12(15.4)	99(33.2)
	Head	18	4(5.1)	14(4.7)
	Teeth	10	1(1.3)	9(3.0)
	Neck	9	0(0.0)	9(3.0)
	Shoulder	9	4(5.1)	5(1.7)
	Arms	0	0(0.0)	0(0.0)
	Hands	4	1(1.3)	3(1.0)
	Chest	21	7(9.0)	14(4.7)
	Abdomen	23	6(7.7)	17(5.7)
	Back	13	3(3.8)	10(3.4)
	Waist	20	2(2.6)	18(6.0)
	Hips	3	2(2.6)	1(0.3)
	Legs	13	2(2.6)	11(3.7)
	Combined pain of two sites	54	15(19.2)	39(13.1)
	Combined pain of three sites	34	10(12.8)	24(8.1)
	Combined pain of four sites	12	1(1.3)	11(3.7)
	Combined pain of five sites	6	4(5.1)	2(0.7)
	Combined five or more painful sites	16	4(5.1)	12(4.0)
**Grading, N(%)**				
	Grade 0	294	55(70.5)	239(80.2)
	Grade 1	17	2(2.6)	15(5)
	Grade 2	15	2(2.6)	13(4.4)
	Grade 3	50	19(24.4)	31(10.4)

### 3.2 Internal consistency reliability

Table 2 displays the Cronbach’s α coefficient values for both the overall and individual items of the C-GCPS-R. The removal of any item from the C-GCPS-R did not significantly increase the Cronbach’s α coefficient, which remained above the recommended value of 0.80. The correlation coefficients between each item of the C-GCPS-R scale and the overall scale ranged from 0.711 (item 1) to 0.929 (item 5), all of which exceeded the recommended value of 0.7. Table 3 presents the correlation between items, PEG scale and pain grades. The results demonstrate strong homogeneity among the items and between the items and the scale, indicating high internal consistency of the total scale.

**Table 2:** Item-Total statistics of the C-GCPS- R.

	Scale Mean if Item Deleted	Scale Variance if Item Deleted	Corrected Item-Total Correlation	Squared Multiple Correlation	Cronbach’s Alpha if Item Deleted
A1	7.51	62.372	0.711	0.621	0.918
A2	7.72	61.966	0.770	0.672	0.915
A3	6.07	37.766	0.935	0.880	0.837
A4	6.19	35.985	0.941	0.904	0.839
A5	6.34	37.456	0.929	0.879	0.840
**Factor 1**					
A3	4.39	24.757	0.932	0.870	0.963
A4	4.50	23.133	0.949	0.900	0.950
A5	4.65	24.342	0.935	0.878	0.960
**Factor2**					
A1	0.73	0.586	0.754	0.569	
A2	0.95	0.620	0.754	0.569	
**C-GCPS-R(total)**: Cronbach’s Alpha 0.944					

*Note*: C-GCPS- R: Graded Chronic Pain Scale-Revised—Simplified Chinese version.

**Table 3:** Interitem correlation statistics of the C-GCPS- R.

	A1	A2	A3	A4	A5	PEG^*a*^	Grade^*b*^
**A1**	1.000						
**A2**	0.754^*c*^	1.000					
**A3**	0.700^*c*^	0.729^*c*^	1.000				
**A4**	0.655^*c*^	0.735^*c*^	0.924^*c*^	1.000			
**A5**	0.659^*c*^	0.720^*c*^	0.905^*c*^	0.929^*c*^	1.000		
**PEG**	0.689^*c*^	0.749^*c*^	0.969^*c*^	0.978^*c*^	0.971^*c*^	1.000	
**Grade**	0.748^*c*^	0.749^*c*^	0.593^*c*^	0.614^*c*^	0.615^*c*^	0.625^*c*^	1.000

*Note*: ^*a*^ The PEG score is derived from the sum of items 3, 4, and 5.

^*b*^ Grade is calculated using the C-GCPS-R grading algorithm.

^*c*^ Correlation is significant at the 0.01 level (two-tailed).

### 3.3 Validity

#### 3.3.1 Content validity

The cultural adaptation of the C-GCPS-R was conducted twice by cultural debugging experts. The first cultural adaptation results showed that the S-CVI of the scale was 0.96, which is greater than 0.9, and the I-CVI was greater than 0.78; however, two experts scored item 2 below 3 points. Therefore, the study continued to culturally adapt the C-GCPS-R, and the second cultural adaptation results showed that the S-CVI of the scale was 1.00, which is greater than 0.9, and the I-CVI was greater than 0.78. The content validity of the scale was excellent. Except for item 6, the correlation coefficients between the other items and the total scale were greater than 0.4 (P<0.01) (see S1 Table).

#### 3.3.2 Construct validity

*3*.*3*.*2*.*1 Exploratory factor analysis*. The EFA results indicate a KMO value of 0.861, which exceeds the recommended threshold of 0.8, and an approximate chi-square value of 553.98 (P<0.01) for the Bartlett sphericity test, indicating that there were common factors among the variables and supporting the use of factor analysis. Using principal component analysis and rotation, two common factors were obtained, accounting for a cumulative total variance explained of 95.303%, which exceeds the recommended thresholds of 85%. The component matrix showed that all items had loadings greater than 0.4 on their respective dimensions. According to the factor loadings, the first common factor consisted of three items, namely, item 3 (loading = 0.869), item 4 (loading = 0.898), and item 5 (loading = 0.906). The second common factor consisted of two items, namely, item 1 (loading = 0.917) and item 2 (loading = 0.756). (Table 4) In the original English version, items 3, 4, and 5 constitute the PEG scale, which corresponds to the item contained in the first common factor obtained in this study. Items 1 and 2 in the second common factor are used to identify HCIP, which is also consistent with the logic of the original scale.

**Table 4:** Factor loadings of the C-GCPS- R.

Item	1	2
**A1**		0.917
**A2**		0.756
**A3**	0.869	
**A4**	0.898	
**A5**	0.906	
**Eigenvalues**	85.867	9.436
**Percentage of variance**	56.648	38.655
**Cumulative percentage of variance**	56.648	95.303

**Fig 1:**
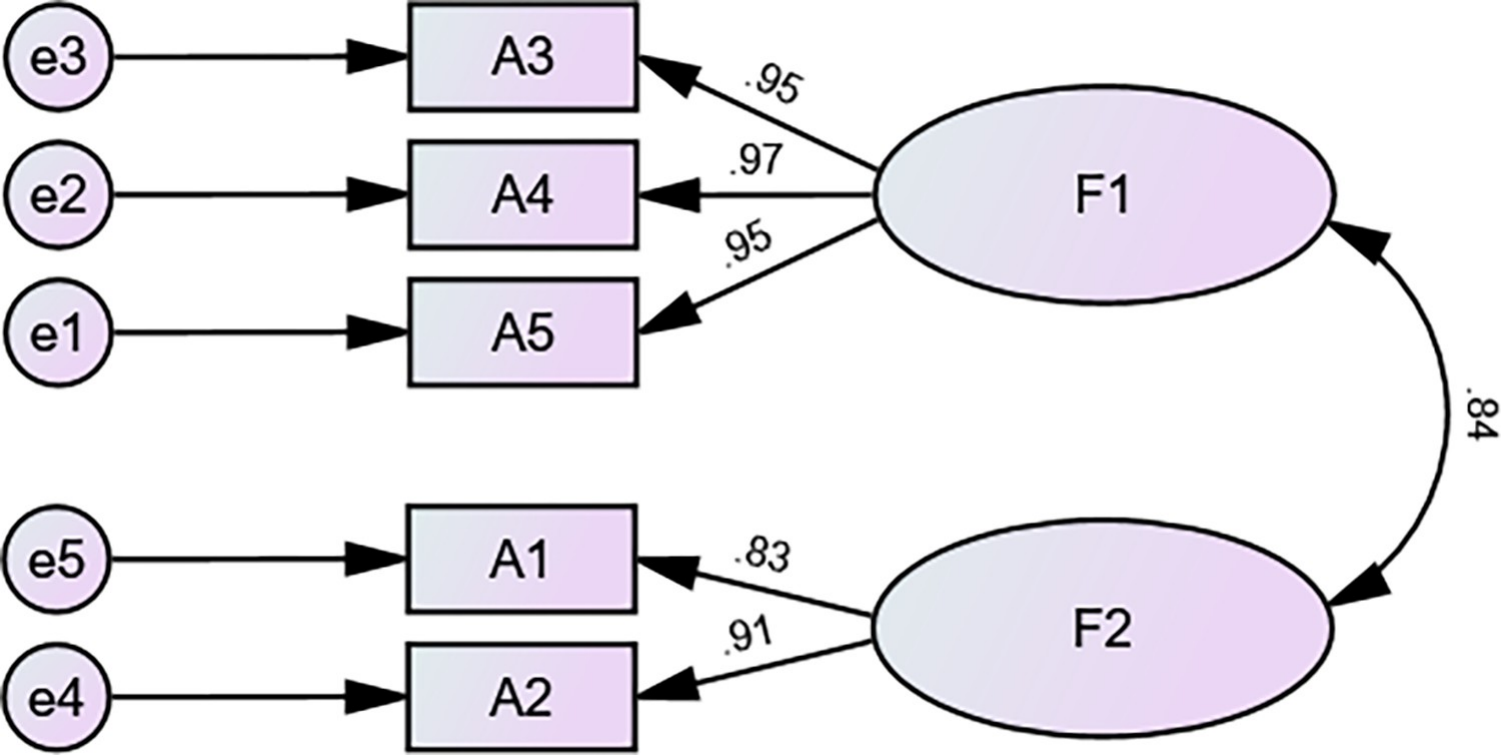
Standardized two-factor structural equation model of the C-GCPS- R. F1, PEG scale; F2, Identify HCIP.

*3*.*3*.*2*.*2 Confirmatory factor analysis*. The CFA was conducted on 298 samples of sample 2 using AMOS 26.0 software to further verify the compatibility between the structure of the C-GCPS-R and the original English version. Based on the dimension of the GCPS-R, the study established two latent variables, identifying the HCIP and PEG subscales, and set five items as observed variables to establish a predictive model for CFA. In this model, X^2^/df is 3.799, RMR is 0.022, AGFI is 0.926, GFI is 0.980, TLI is 0.984, CFI is 0.994, and RMSEA is 0.097. All indicators were within the recommended range, indicating a good model fit. The model fit indicators and the standardized two-factor structural equation model are shown in Table 5 and Fig 1.

**Table 5:** Model fit statistics for the C-GCPS- R.

Model	Chi-square/df	GFI	RMR	AGFI	TLI	CFI	RMSEA
**Model fit index**	3.799	0.980	0.022	0.926	0.984	0.994	0.097
**Recommended value**	<5	>0.8	<0.05	>0.8	>0.9	>0.8	<0.1

*Note*: X^2^/df: Chi-square freedom ratio; GFI, Goodness of fit index; RMR, Root Mean Square Residual; AGFI, Adjusted goodness of fit index; TLI, Tucker-Lewis Index; CFI, comparative fit index; RMSEA, root mean square error of approximation`

According to Table 6, the C.R. values for each dimension are above 0.70, and the AVE values are above 0.50. This indicates that the C-GCPS-R dimensions have good convergent validity, meaning that each item is able to reflect its corresponding dimension well.

**Table 6:** Convergent validity.

Factor	Item	Estimate	S.E.	C.R.	P	AVE	C.R.
**F1**	A3	0.952	0.026	37.274	***	0.92	0.97
**F1**	A4	0.971	0.026	41.424	***		
**F1**	A5	0.954					
**F2**	A1	0.831	0.054	17.464	***	0.76	0.86
**F2**	A2	0.907					

*Note*: ***: p < 0.001(two-tailed); AVE: Average variance extracted; C.R.: Composite Reliability.

Table 7 demonstrates that the square root of AVE for both common factor 1 and common factor 2 exceeds the correlation coefficients between these factors and other factors, indicating a strong discriminant validity between the two common factors.

**Table 7:** Discriminant validity.

	F1	F2
**F1**	**0.96**	
**F2**	0.84	**0.87**

*Note*: Square root of AVE in bold on diagonals.

The NRS was used as the calibration tool of the C-GCPS-R. The mean scores of the PEG scale were highly correlated with those of the NRS, with a correlation coefficient of 0.969 and a significance level of P<0.01 (Table 8).

**Table 8:** Criterion correlation validity.

	NRS	PEG mean score ^*a*^
**NRS**	1	
**PEG mean score**	.969**	1

*Note*: **. Correlation is significant at the 0.01 level (2-tailed).

^*a*^ Average score of each item in PEG scale.

**Fig 2:**
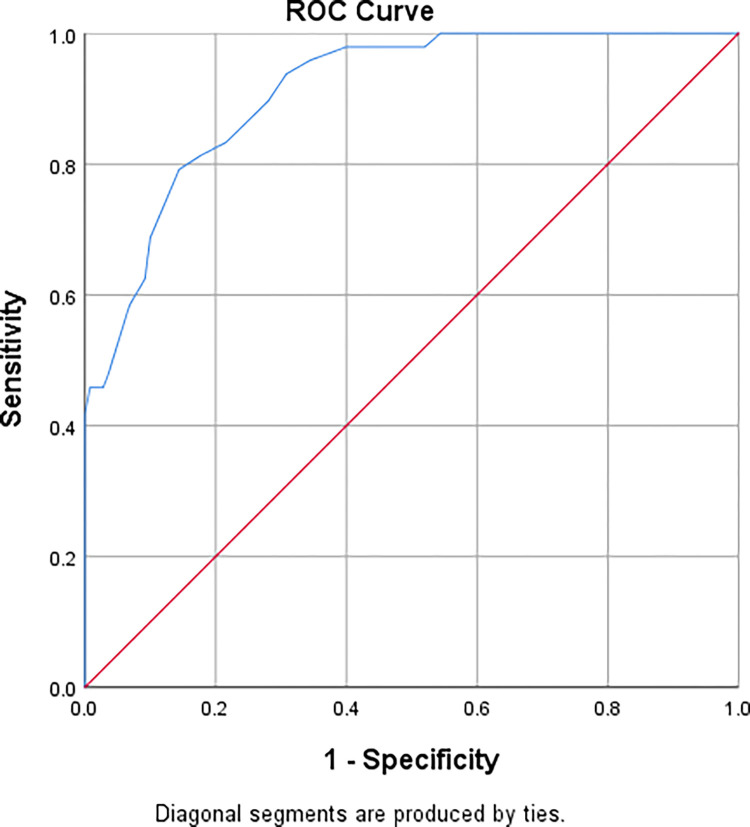
The ROC of the C-GCPS-R based on item 6. The sensitivity was 81.3% and the specificity was 82.4%. The area under curve was 0.91 (95% [CI] 0.87–0.95).

*3*.*3*.*2*.*3 Diagnostic accuracy*. Furthermore, with regard to the relationship between chronic pain and participants’ work ability, we conducted binary logistic regression analysis. The results indicated a significant impact of chronic pain on participants’ work ability (P<0.001). (Table 9) Therefore, we plotted ROC for variables and determined that a chronic pain score of greater than 11 had the strongest predictive accuracy for work impact (see S2 Table). The sensitivity and specificity were found to be 81.3% and 82.4%, respectively, and the area under the curve was 0.91 (95% confidence interval [CI] 0.87–0.95), which was consistent with the predictive effect of chronic pain at GCPS-R grade 2 (≥12 points) or higher on work impact (Fig 2).

**Table 9:** Binary logistic regression analysis of item 6 and chronic pain.

Independent Variables	*Ba*	SE_*b*_	Wald	Exp(B)	Sig.
**Cp**	1.544	.333	21.485	4.682	.000
**Constant**	-2.150	.216	99.347	.117	.000

*Note*: Cp: Chronic pain; SE, Standard Error.

## 4 Discussion

In this paper, we report the successful translation and cross-cultural adaptation of the GCPS-R. We observed a high completion rate of the scale, indicating high levels of comprehensibility and acceptability among participants. We found the C-GCPS-R to be reliable and valid in grading chronic pain among Chinese participants.

We ensured equivalence with the English version through two cross-cultural adaptations, each involving a panel of seven experts with diverse research backgrounds. The resulting simplified Chinese version of the C-GCPS-R was found to be content valid. This development may greatly benefit the assessment of pain severity grading among the Chinese population.

After conducting correlation analysis on the scale, we found that, except for item 6, the correlation coefficients between each item of the C-GCPS-R and the total scale were greater than 0.4 (P<0.01), indicating that each item is both interrelated and has good discriminant validity. In the Turkish version of the GCPS-R psychometric assessment, analysis of item 6 was also eliminated [27]. This is because item 6 in the original English version was mainly used to confirm the impact of chronic pain on participants’ work, and adding this item clarified the clinical significance of chronic pain without affecting the severity grading of chronic pain [25], resulting in a low correlation with item 6. In subsequent psychometric validation, this item was not included in this study.

The overall internal consistency α coefficient of the C-GPS-R was high at 0.944, which has also been observed in other scales translated from English to simplified Chinese [39–41]. In addition, the internal consistency α coefficient of the C-GCPS-R was higher than that of the Italian version (α = 0.86) [42], German version (α = 0.82) [43], and Turkish version (α = 0.903) [27]. These results indicate that the GCPS-R has good internal consistency and stability when used in different countries and populations, especially for the Chinese population. To further explore the impact of scale items on the quality of the scale, this study calculated the changes in Cronbach’s α (0.792–0.813) after each item was deleted one by one. The results showed that the change was small, which is consistent with the trend of other studies on cultural adaptation of the GCPS-R [27]. Therefore, to ensure the integrity of the original scale structure, the remaining five items were retained to ensure that it could measure the severity grading of chronic pain.

In EFA and CFA, we finally obtained two common factors that were consistent with the factor structure of the original English version and the Turkish version of the GCPS-R [25, 27]. The first common factor was the PEG scale, which serves as a subscale of the GCPS-R and can be used to assess subjects’ average pain severity, enjoyment of life, and interference with general activities. Krebs demonstrated the reliability of the PEG scale in primary care patients with moderate to severe chronic pain, with a reliability of 0.73, and in veterans, with a reliability of 0.89 [44]. The cross-cultural adaptation study of the Turkish version of the GCPS-R by Senturk showed that the reliability of the PEG subscale was 0.90 [27]. In this study, the internal consistency reliability of the PEG subscale was 0.95, which was similar to other research results, indicating that the C-GPS-R has good high reliability. The second common factor in this study was used to identify HCIP, which is defined as pain that is associated with persistent limitations in work, social, and self-care activities [45, 46]. This dimensional approach allows users to identify HCIP through two simple questions [45], providing a very easy and scientific method for grading chronic pain. In Dokyoung’s study on the perception differences of chronic pain between young and older people with HCIP, the same dimensional approach was used to define severe pain, and the different impacts of HCIP on daily life activities were compared between the two groups [47]. The results showed that there were similarities and differences in the areas of pain impact between the two age groups. Both groups of subjects had similar pain impacts on basic physical activities (such as walking and standing) and instrumental daily activities (such as doing housework, driving, and shopping), but young people with HCIP considered work to be the third major activity affected by pain, while the older group ranked social activities, exercise, and sleep as the third most affected. Therefore, the definition of HCIP can not only conveniently, quickly, and effectively identify such patients but also refine the characteristics of the impact of this pain on daily life activities among different populations.

In addition, given the wide application of the NRS in clinical settings and the consistency of scoring rules with single-item scoring rules on the PEG Scale, we applied the NRS and the C-GCPS-R for comparison and tested the criterion validity of the C-GCPS-R. The correlation results showed a highly significant correlation between the average score on the PEG scale and the NRS score, which is consistent with other research results. Boonstra and Gerbershagen’s study indicated that a score of 4 or 5 on a 0–10 scale can distinguish between mild and moderate pain [48, 49], while the PEG scale had a cutoff point of 12, which is consistent with other studies [50, 51]. Therefore, under the premise of consistent evaluation effectiveness with the NRS, this scale provides a multidimensional evaluation of the subject’s pain level and the impact of pain on personal life, greatly enriching the practice of chronic pain assessment and facilitating clinical workers in better understanding the degree of chronic pain in patients and developing targeted interventions.

Given the role of item 6 in the clinical significance of chronic pain, we analyzed the relationship between chronic pain grade and subjects’ work activities (Item 6). The results showed a significant correlation between the two, consistent with other studies [27]. In the original English version, the researchers demonstrated that subjects with chronic pain grade 3 had more activity limitations (such as housework, transportation, and going out) than those with grade 2 chronic pain by comparing the impact of different pain grades on subjects’ daily lives. We also analyzed the sensitivity and specificity of item 6 for subjects with GCPS-R grade 3 and those who self-reported severe pain after evaluating indicators of poor health status, negative coping beliefs, activity limitations, and long-term opioid treatment. The results showed that the GCPS-R grade 3 had a good effect in identifying other HCIP indicators [25, 27]. In the Turkish version of GCPS-R, the authors compared the number of subjects who answered "Yes" to item 6 with those who had a chronic pain grade 3 and found that 84.1% of the subjects who were unable to work belonged to grade 3, indicating that item 6 had better sensitivity and specificity in assisting in identifying grade 3 chronic pain [27]. Both studies clarified the predictive role of specific pain grading on work activity impact using different pain assessment scales. Although this study found good predictability of pain grades 2 and 3 on work impact, further statistical analysis of the factor structure of the scale was limited due to its limited use. Therefore, further research should be conducted in the future.

During the process of assessing the chronic pain grades of the Chinese population, we found that the largest number of participants reported experiencing no pain, which may be attributed to several factors. First, although the C-GCPS-R can be conceptually and semantically interchangeable with the original scale during cultural adaptation, potential translation bias may still exist due to cultural differences between Chinese and Western cultures [52]. Second, ethnicity and culture have an important influence on the expression of pain among individuals [53], and personal attitudes and emotions toward pain may affect the perception of pain intensity. In Chinese Confucianism, the body was often metaphorically socialized as the state and politics in ancient China, and this category has been deeply ingrained in the context of Chinese culture. Therefore, Chinese culture does not encourage individuals to directly express physical and psychological pain, and endurance is considered an important virtue or survival strategy. This feature may greatly affect the expression of pain among the Chinese population [54].

This study has several limitations that should be noted. First, the sample size of this study is relatively limited, as it only includes the population of Changchun, China. Therefore, caution should be exercised when generalizing the findings of this study. Second, since data collection was performed in different hospitals in Changchun, which has high outpatient mobility, the reliability of test-retest reliability has not been confirmed; thus, the stability of the model requires further testing. Third, although the NRS was used as the criterion scale in this study for measuring pain levels, it only serves to distinguish between levels of pain and does not have any clinical evaluative function. Furthermore, other chronic pain assessment scales that have functional impact dimensions were not used to compare and evaluate the relationship between pain grading and work, making it impossible to further distinguish the differences in the impact of the C-GCPS-R grades 2 and 3 on work. In future studies, more diverse subjects should be recruited from different regions, sample size should be expanded to supplement the test-retest reliability of the C-GCPS-R and use more measurement tools related to chronic pain to further provide evidence of its applicability.

## 5 Conclusion

The present study has developed a Simplified Chinese version of the GCPS-R and further examined its validity and reliability. Results indicate that the C-GCPS-R has good psychometric properties and is a useful and reliable tool for assessing chronic pain grades among the Chinese population. Moreover, the C-GCPS-R with only 6 items and a two-factor structure may enhance its usability, making it a better option for clinical applications and scientific research in mainland China. However, further research is needed to improve the pain assessment properties of the C-GCPS-R.

## Supporting information

S1 File English version of the C-GCPS-R. (PDF)

S1 Table Correlation of each item with the total scale of the C-GCPS-R. (DOCX)

S2 Table The results of the receiver operating characteristic curve analysis using impact work as a standard. (DOCX)

S1 Data (ZIP)
