# Supplementary material for: The cross-cultural adaptation and psychometric properties of the Graded Chronic Pain Scale-Revised—Simplified Chinese version
Source: PLoS One. 2023 Oct 10;18(10):e0292747. doi: 10.1371/journal.pone.0292747 (PMC10564124; doi:10.1371/journal.pone.0292747)
Supplement: S1 File — (PDF) [file pone.0292747.s001.pdf]

# The cross-cultural adaptation and psychometric properties of Graded Chronic Pain Scale-Revised - Simplified Chinese version

**S1 File** English version of C-GCPS-R

1. In the past 3 months, how often did you have pain?

☐Never ☐Some days ☐Most days ☐Every day

**If you never had pain in the past 3 months, skip the remaining questions.**

2. Over the past 3 months, how often did pain limit your life or work activities?

☐Never ☐Some days ☐Most days ☐Every day

**Now think about pain you have had during the past 7 days**

3. What number best describes your pain, on average?

☐0（No pain） ☐1 ☐2 ☐3 ☐4 ☐5 ☐6 ☐7 ☐8 ☐9 ☐10（Pain as bad as you can imagine）

4. During the past 7 days, what number best describes how pain has interfered with your enjoyment of life?

☐0（Does not interfere） ☐1 ☐2 ☐3 ☐4 ☐5 ☐6 ☐7 ☐8 ☐9 ☐10（Completely interferes）

5. During the past 7 days, what number best describes how pain has interfered with your general activity?

☐0（Does not interfere） ☐1 ☐2 ☐3 ☐4 ☐5 ☐6 ☐7 ☐8 ☐9 ☐10（Completely interferes）

6. Are you not working or unable to work due to pain or a pain condition?

☐Yes ☐No
