# Supplementary material for: The cross-cultural adaptation and psychometric properties of the Graded Chronic Pain Scale-Revised—Simplified Chinese version
Source: PLoS One. 2023 Oct 10;18(10):e0292747. doi: 10.1371/journal.pone.0292747 (PMC10564124; doi:10.1371/journal.pone.0292747)
Supplement: S1 Table — (DOCX) [file pone.0292747.s002.docx]

The cross-cultural adaptation and psychometric properties of Graded Chronic Pain Scale-Revised - Simplified Chinese version

**S1 Table** Correlation of each item with the total scale of the C-GCPS-R

| Item | R_s_ | P-value(two-tailed) |
| --- | --- | --- |
| A1 | 0.748 | P＜ 0.01 |
| A2 | 0.749 | P＜ 0.01 |
| A3 | 0.593 | P＜ 0.01 |
| A4 | 0.614 | P＜ 0.01 |
| A5 | 0.615 | P＜ 0.01 |
| A6 | **0.361** | P＜ 0.01 |

*Note:* C-GCPS- R: Graded Chronic Pain Scale-Revised - Simplified Chinese version; R_s:_ correlation between the item and the total scale; Bolded for Rs < 0.4.
