# Supplementary material for: The cross-cultural adaptation and psychometric properties of the Graded Chronic Pain Scale-Revised—Simplified Chinese version
Source: PLoS One. 2023 Oct 10;18(10):e0292747. doi: 10.1371/journal.pone.0292747 (PMC10564124; doi:10.1371/journal.pone.0292747)
Supplement: S2 Table — (DOCX) [file pone.0292747.s003.docx]

The cross-cultural adaptation and psychometric properties of Graded Chronic Pain Scale-Revised - Simplified Chinese version

**S2 Table** Results of the receiver operating characteristic curve analysis using impact work as a standard

| PEG Cutoff Points | Youden Index, % | Sensitivity, % | Specificity, % |
| --- | --- | --- | --- |
| 0.5 | 0.376 | 1 | 0.376 |
| 1.5 | 0 | 1 | 0 |
| 2.5 | 0.459 | 0.979 | 0.48 |
| 3.5 | 0 | 0.979 | 0.021 |
| 4.5 | 0.555 | 0.979 | 0.576 |
| 5.5 | 0 | 0.979 | 0.021 |
| 6.5 | 0.614 | 0.958 | 0.656 |
| 7.5 | 0 | 0.938 | 0.063 |
| 8.5 | 0.616 | 0.896 | 0.72 |
| 9.5 | 0 | 0.833 | 0.167 |
| **10.5** | **0.637** | **0.813** | **0.824** |
| 11.5 | 0 | 0.792 | 0.208 |
| 12.5 | 0.588 | 0.688 | 0.9 |
| 13.5 | 0 | 0.625 | 0.375 |
| 14.5 | 0.515 | 0.583 | 0.932 |
| 15.5 | 0 | 0.479 | 0.521 |
| 16.5 | 0.43 | 0.458 | 0.972 |
| 17.5 | 0 | 0.458 | 0.542 |
| 18.5 | 0.417 | 0.417 | 1 |
| 20 | 0 | 0.396 | 0.604 |
| 21.5 | 0.354 | 0.354 | 1 |
| 22.5 | 0 | 0.25 | 0.75 |
| 23.5 | 0.229 | 0.229 | 1 |
| 25 | 0 | 0.188 | 0.813 |
| 26.5 | 0.125 | 0.125 | 1 |
| 28.5 | 0 | 0.104 | 0.896 |

*Note:* PEG: Pain, Enjoyment, and General Activities Scale; Bolded indicates best cut-off score.
